# Screening for cold tolerance resources in maize seedlings and analysis of leaf cell responses

**DOI:** 10.3389/fpls.2025.1565831

**Published:** 2025-06-25

**Authors:** Mengting Hu, Dan Zhang, Wentao Du, Huijuan Tian, Ying Hao, Shuqi Ding, Kaizhi Yang, Ruohang Xu, Lei Zhang

**Affiliations:** ^1^ College of Agriculture, Tarim University, Alar, China; ^2^ Key Laboratory of Genetic Improvement and Efficient Production for Specialty Crops in Arid Southern Xinjiang of Xinjiang Corps, College of Agriculture, Tarim University, Alar, China

**Keywords:** maize, seedling stage, low temperature, photosynthetic physiology, cell, screening

## Abstract

Using 63 maize varieties as materials, this study employed indoor low-temperature stress testing methods to evaluate cold tolerance during the seedling stage. The aim was to investigate the low-temperature resistance of different maize varieties during the seedling stage and to conduct photosynthetic physiological analysis and leaf cell responses for extreme materials. The results of the experiment indicate that: (1) Different maize varieties exhibit variations in cold tolerance during the seedling stage, and the extent to which various measurement indicators are affected by low-temperature stress differs. (2) The comparative analysis of phenotypic traits and physiological indexes showed that low temperature stress inhibited the growth of plant height and stem diameter, and had a great impact on leaf width and leaf area. The relative conductivity and MDA content showed an upward trend and showed a very significant difference. (3) Correlation analysis shows that phenotypic traits and physiological indicators under normal temperature and low-temperature stress have different correlations. (4) Principal component analysis (PCA) transformed the 12 measurement indicators into 7 independent indicators, with a cumulative contribution rate of 87.12%. Leaf area, SOD, stem thickness, relative conductivity, proline content, CAT, and chlorophyll content were identified as the primary evaluation indicators for cold resistance in maize seedlings. (5) Using the membership function method combined with cluster analysis, the cold tolerance of the 63 maize varieties was classified into five categories: extremely strong cold tolerance, strong cold tolerance, moderate cold tolerance, weak cold tolerance, and cold-sensitive. Based on the ranking by D value, two extreme materials were identified: the variety with extremely strong cold tolerance is No. 11 (Jiuyang 818), and the cold-sensitive variety is No. 6 (JR288). (6) Phenotypic observations and measurements of photosynthetic physiological indicators in the two extreme materials revealed that the variety with extremely strong cold tolerance, Jiuyang 818, exhibited more robust plant growth and stronger photosynthetic capacity. (7) Cytological observations of maize leaves revealed that Jiuyang 818 exhibited best cold tolerance during the low temperature stress phase, but JR288 showed significant wilting of leaves six days after low-temperature stress. This finding is consistent with the phenotypic observations and photosynthetic physiological determination results obtained in the preliminary studies. Through indoor identification methods, this study screened and characterized different maize varieties to identify cultivars with varying levels of low-temperature tolerance. The research elucidates the effects of low-temperature stress on photosynthetic physiology and associated changes in leaf cellular structure. These results may provide theoretical references for future studies on low-temperature stress tolerance.

## 1 Introduction

Maize (Zea mays. L) is the leading cereal crop in China and serves as a crucial source of food, feed, energy, and industrial raw materials (Zhang et al., 2024a; Gu et al., 2024). With the rapid development of the domestic maize industry, its planting area has continuously expanded. Due to global climate change, extreme weather events can adversely affect the early growth stages of maize and even lead to plant death. Abiotic environmental factors such as high temperatures, low temperatures, drought, and salinity significantly limit the growth and development of maize (Zargar et al., 2024). Among environmental stress factors, low temperature is one of the most detrimental stresses encountered by higher plants (Theocharis et al., 2012; Zhou et al., 2017), and it exerts a significant impact on plant productivity and geographical distribution (Guo et al., 2017; Liu and Zhou, 2017; Shi et al., 2018). To cope with low-temperature stress, most temperate plants have evolved complex cold adaptation mechanisms to mitigate the damage caused by chilling injury. However, many crop species lack the ability to adapt to cold conditions and are sensitive to cold stress, often resulting in significant production losses due to chilling injury. This is particularly true for plants originating from tropical and subtropical regions (Sugimoto et al., 2004; Zhang et al., 2004). The seedling stage of maize primarily refers to the period from the end of germination to the three-leaf stage. This phase is crucial for the establishment of plant morphology and the determination of yield and it is also a period of sensitivity to low temperatures (Xuhui et al., 2022). The morphological construction and biomass accumulation during the seedling stage are critical factors influencing plant yield and quality. When maize seedlings encounter low temperatures during this period, it significantly affects the subsequent accumulation of organic matter, severely restricting the subsequent development and yield of the maize (Yi et al., 2021; Zhao et al., 2019). Wang et al. proposed that while C4 crops, such as maize, have high photosynthetic potential, this potential is constrained by low temperatures, preventing the full expression of their photosynthetic capabilities. Specifically, under severe temperature drops, maize yield reductions can exceed 15% (Wang et al., 2008). Strigens et al. found that the morphological indexes related to plant height, fresh weight, leaf number and leaf area of maize were affected to varying degrees under low temperature stress (Strigens et al., 2013). At the same time, under the influence of low temperature, the dry and fresh weight, branch number and root morphology of maize seedling roots changed (Kaspar and Bland, 1992). Cofer et al. showed that low temperature stress led to the yellowing and wilting of maize leaves, and the leaf length, leaf area and leaf unfolding efficiency were significantly reduced (Cofer et al., 2018).

Plants will experience physiological and cellular changes when exposed to low temperature stress. Therefore, plants need to maintain cellular normal function and activity, especially the stability of the cell membrane and the structure of biologically active proteins, in order to survive in harsh environments. These physiological changes include alterations in cell membrane structure, enhanced antioxidant defense capabilities, and modifications in osmoregulatory substances (Guo et al., 2018). Low temperatures can cause damage to plant cell membranes, increase membrane permeability, and lead to the outward leakage of substances and electrolytes within the cell membrane (Orvar et al., 2000; Hussain et al., 2018). Relative electrical conductivity and MDA content are indicators of cell membrane damage. The peroxidation of the cell membrane generates MDA, while high electrical conductivity represents damage to the cell membrane permeability, with severe electrolyte penetration (Sun et al., 2020; Meng and Sui, 2018). Among them, the content of malonaldehyde (MDA), hydrogen peroxide and relative conductivity will be significantly higher under low temperature stress (Zuo et al., 2022b). Under low-temperature conditions, the MDA content in maize leaves increases; however, the increase is less pronounced in cold-tolerant varieties compared to cold-sensitive varieties. In addition, the MDA content of cold-sensitive maize varieties was higher than that of cold-tolerant maize (Meng et al., 2022). Research suggests that corn varieties with strong cold resistance have less cell membrane damage and a lower degree of increase in relative electrical conductivity and MDA content (Hu et al., 2008; Lainé et al., 2023; Yu et al., 2024). In addition, the reason for the difference in low temperature tolerance of different genotypes of maize may be related to the antioxidant system. The activity of antioxidant enzymes such as SOD, POD and CAT was increased more in varieties with strong cold tolerance (Zhu et al., 2015).

Additionally, low temperatures can affect osmotic pressure changes in plants, leading to dehydration of cells and tissues (Kazemi-Shahandashti and Maali-Amiri, 2018; Laishram et al., 2024). Proline concentration serves as an osmoprotectant in plants under cold stress, responsible for maintaining membrane integrity to prevent cellular dehydration, playing a crucial role in resisting cold stress (Zuo et al., 2022a). Many studies have shown that the accumulation of proline is positively correlated with the improvement of plant stress resistance (Sadeghipour, 2020; Misbah Qirat et al., 2018; Elewa et al., 2017). Proline content accumulates in varieties with strong cold resistance (Zhao et al., 2022), and the proline content increased with the longer the low temperature stress time, so as to alleviate the damage of low temperature stress to maize (Jiao et al., 2022). For varieties with weak resistance to low temperature, the significant increase of proline content in the later stage may be caused by the damage caused by low temperature stress.

Leaves are mainly responsible for regulating photosynthesis and transpiration of plants (Zhang et al., 2024b). Chlorophyll is one of the main chloroplast components of photosynthesis. Research shows that low temperature stress can cause an irreversible decrease in the photosynthetic rate of plants, and the relative content of chlorophyll is positively correlated with the photosynthetic rate (Aroca et al., 2001). Under continuous low temperature conditions, the net photosynthetic rate, stomatal conductance and transpiration rate all decreased continuously (Bhattacharya, 2022a). In plant cold resistance research, morphological anatomical structural characteristics are often used as important reference or identification indicators (Yavas et al., 2024; Soltabayeva et al., 2021). Under low temperature stress, leaf thickness, sponge tissue thickness, fence tissue thickness and other indexes decreased (Cao et al., 2014; Dong et al., 2025). Additionally, plants can enhance their resistance to adverse environments by increasing the thickness of the cuticle, epidermis, and leaf blades (Rape, 1999; Equiza et al., 2001; Cai and Song, 2001). Under low temperature stress, the structure of chloroplasts was partially destroyed and the number decreased. The structure of the chloroplast membrane is arranged in a disordered and distorted manner, the cells cavitate and disintegrate, and the plasma membrane is damaged and blurred (Zhang Jing and Zhu WeiMin, 2012; Wen et al., 2014; Venzhik et al., 2014). Bilska-Kos et al. showed that cold treatment resulted in an increase in leaf thickness and mesophysial cell layer thickness of cold-tolerant maize strains, while the opposite was true of cold-sensitive maize strains (Venzhik and Moshkov, 2023; Bilska-Kos et al., 2018).

As the impact of global warming becomes more pronounced, extreme weather events become more frequent, and the impact of cold weather on crops becomes more and more unstable. As the second most important food crop in China, corn plays a pivotal role in agriculture, economy and energy. Under the appropriate temperature, corn can grow and develop normally, providing a good foundation for the arrival of the filling period. However, when corn is subjected to low temperature stress in the seedling stage, it will affect the normal growth and development of corn plants, and in serious cases, it will lead to the death of corn seedlings, causing huge losses to agricultural production. The purpose of this study was to investigate the plant performance, physiological index changes and the effect of low temperature on leaves of maize under low temperature stress at seedling stage, classify the maize varieties for cold tolerance, clarify the performance of maize varieties to low temperature, and provide theoretical basis for the utilization of maize germplasm resources in the later stage.

## 2 Materials and methods

### 2.1 Materials

A total of 63 corn varieties currently promoted in China were selected as experimental materials. The varieties are mainly from Beijing province, Jilin province, Inner Mongolia, Gansu province, Henan province, Jiangsu province, etc. These germplasm resources have rich diversity in genetic background and germplasm source, which lays a solid foundation for subsequent low-temperature response research and germplasm screening. Specific information is shown in **Table 1**.

**Table 1:** Names and sources of 63 maize varieties.

Code	Varity	Province	Code	Varity	Province
1	Sheng Yu 908	Gansu province	33	Run Min 759	Jilin province
2	Shan Ke No.9	Anhui province	34	Heng Yu 369	Jilin province
3	Ke Yu 192	Jilin province	35	Qin Dan 969	Liaoning province
4	Nong Hua 032	Beijing province	36	Simon 668	Inner Mongolia
5	De Yu 125	Inner Mongolia	37	Gan Xin 2818	Gansu province
6	JR288	Inner Mongolia	38	Jin Kai No.1	Gansu province
7	Jin Feng Jie 607	Beijing province	39	Dun Yu 15	Gansu province
8	Simon M1573	Inner Mongolia	40	Liang Yu 99	Shandong province
9	Simon M1618	Inner Mongolia	41	Tian Yu 819	Jilin province
10	Ning Yu 688	Jiangsu province	42	Rui Pu909	Shanxi province
11	Jiu Yang 818	Gansu province	43	Fu Di 201	Gansu province
12	You Qi 666	Jilin province	44	Jin Dan No. 73	Beijing province
13	Jin Kai No.3	Gansu province	45	Dun Yu 606	Gansu province
14	Tian Yu 108	Jilin province	46	DK117	Beijing province
15	Sheng Yu 906	Gansu province	47	Da Feng No. 14	Shanxi province
16	Sheng Yu 963	Shaanxi province	48	Zhong Yu 968	Shaanxi province
17	Jing Ke 968	Beijing province	49	Jin Run 919	Liaoning province
18	Jiu 623	Gansu province	50	Dun Yu 260	Gansu province
19	Jin Kai No.5	Gansu province	51	Simon 3358	Inner Mongolia
20	Zhen Dan 958	Henan province	52	Ning Dan No. 19	Gansu province
21	Bosch Nong	Gansu province	53	Hong Xing 990	Jilin province
22	Jiu 516	Gansu province	54	Jin Qing No. 1	Jilin province
23	Jin Yuanzao 247	Gansu province	55	He Yu 187	Xinjiang
24	Ken Yu 90	Gansu province	56	Liang Yu 88	Shandong province
25	Ye Feng 203	Gansu province	57	Sheng Mei 999	Beijing province
26	De Yi 366	Gansu province	58	Xiny Yin KX3564	Shanxi province
27	Jiu Shenghe 257	Xinjiang	59	Feng Yu No. 3	Gansu province
28	Ji Yu 516	Gansu province	60	Wo Yu No. 3	Hebei province
29	Ao Mei 95	Gansu province	61	Liang Yu 918	Shandong province
30	Bi Xiang 638	Beijing province	62	Xian Yu 128	Liaoning province
31	Ning Yu 468	Jiangsu province	63	Tie 391	Sichuan province
32	You Qi 909	Jilin province			

### 2.2 Experimental designs

#### 2.2.1 Laboratory seedling test

Maize seeds of full growth and uniform size were selected, disinfected with 75% alcohol for 3 minutes, and rinsed three times with distilled water. Germination was induced for 24 hours. Seeds exhibiting consistent root and bud lengths were chosen and planted in outdoor potted plants (pot dimensions: 21 cm diameter and 20 cm height). Each pot contained 10 seedlings, and each treatment was replicated three times. Upon reaching the triloba phase (10–12 days), the samples were transferred to an RTOP-1000Y intelligent artificial climate incubator for low-temperature stress treatment at 5°C (day/night). The photoperiod was set to 14 hours of light and 10 hours of darkness, with a light intensity level of 3 and humidity maintained at 40%.

The experimental design included a control group (0 days, CK), low-temperature stress groups for 3 days (Temperature1, T1) and 6 days ((Temperature2, T2), and a recovery group exposed to outdoor temperatures for 3 days after low-temperature stress (Temperature Recover, Tr). For each treatment, leaf samples were collected, cut into small pieces, thoroughly mixed to minimize variability, and immediately immersed in liquid nitrogen for subsequent analysis of relevant physiological and biochemical indicators.

#### 2.2.2 Selection of stress time points

**Figure 1:**
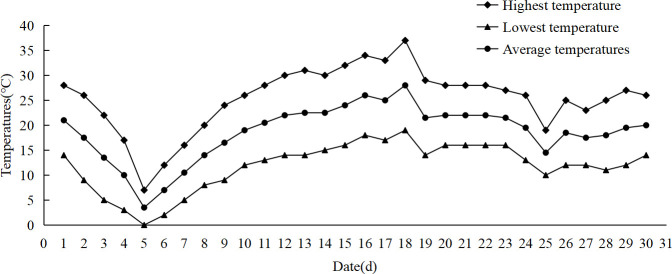
Temperature change in May 2023.

**Figure 2:**
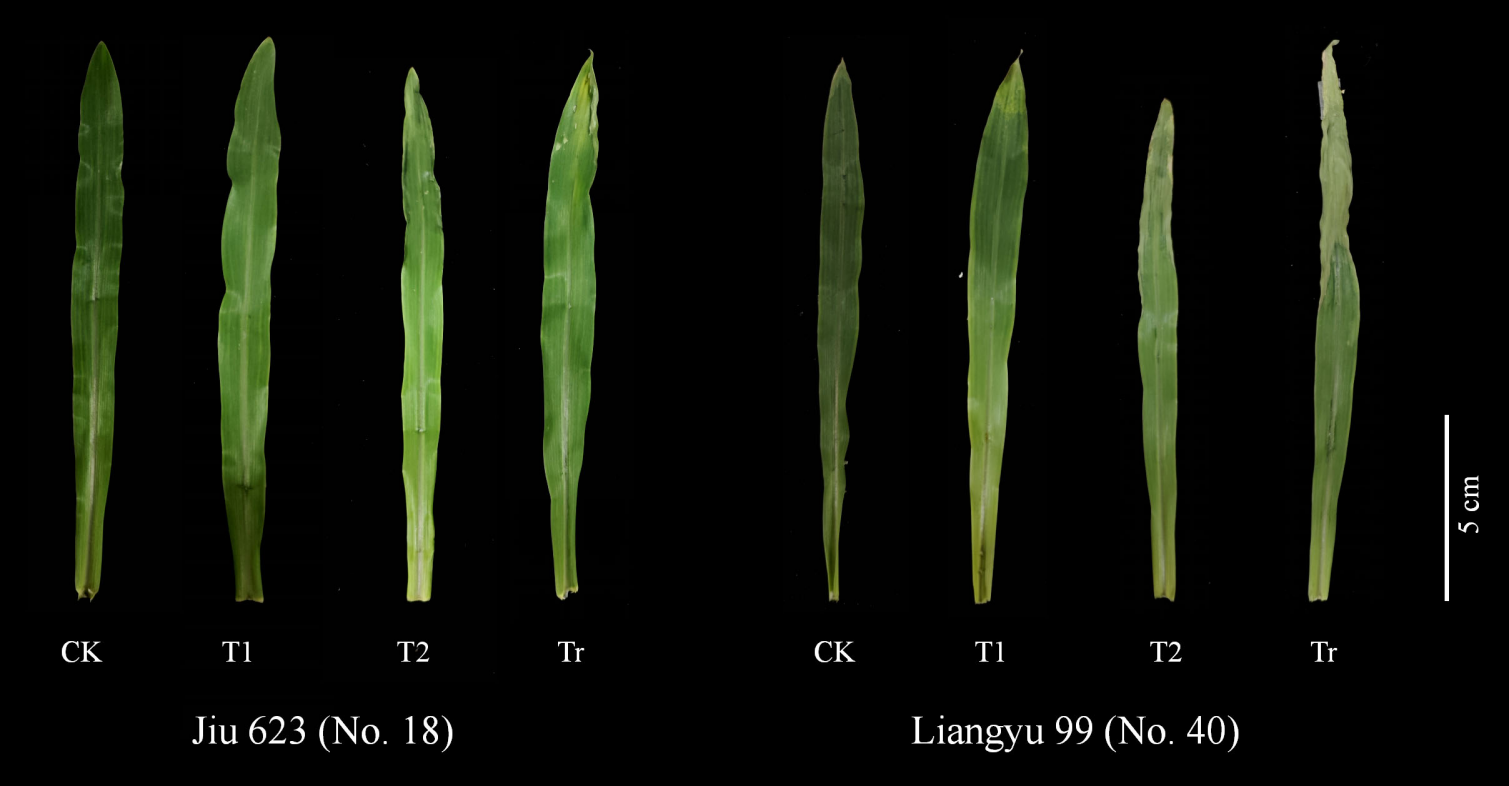
Changes in leaves of some varieties under different treatments at 5°C temperature.

The seedling stage of maize is a critical period for growth and development, and it is sensitive to low temperature. The response mechanism and adaptability of maize to low temperature during the sensitive period could be studied by conducting low temperature stress experiments for different durations at the seedling stage. This experiment’s stress time point is mainly based on the following reasons:

(1) In May 2023, a week-long low temperature occurred at the test site (Alar City, Xinjiang), resulting in frost damage to cotton and corn that year (**Figure 1**). (2) In the later stage of indoor low temperature stress, it was found that there was no significant phenotypic difference between maize seedlings at 7°C and 9°C, especially the leaf damage. When the temperature was set to 5°C, there was a difference in the phenotype of the seedlings. The main results were that after 3 days of low temperature stress, the leaves of most varieties of maize were significantly damaged. After 6 days of low temperature stress, more than half of the maize varieties had significant differences in leaf changes and overall plant performance (**Figure 2**). (3) Under the observation of the above temperature, it was found that the low temperature stress was too short (1–2 d), and the changes of seedlings could not be observed. When the low temperature time is too long (7–9 d), the seedlings are at risk of freezing to death when the ability to resist low temperature is exceeded, and the recovery ability of the plant is not good during the recovery stage at room temperature. The response of maize seedlings to low temperature stress can be comprehensively studied from multiple perspectives, including short-term and long-term adaptation mechanism, injury degree and recovery ability, and provide a theoretical basis for cold resistance breeding of maize.

#### 2.2.3 Observation of the microstructure of leaves

1. Selection of materials: Based on the cold resistance screening of 63 maize varieties at seedling stage, two extreme cold resistance materials were obtained: the very strong cold resistance variety and the cold sensitive variety.
2. Sampling position: When the three leaves are at the same stage, select the second fully unfolded leaf, 3-4cm away from the tip of the leaf, choose the same part of each plant as far as possible, and cut into 10mm long strips (Liu, 2022).
3. Sampling period: the outdoor temperature was the control (CK). There is 3 days of low temperature stress (Temperature1, T1), 6 days of low temperature stress (Temperature1, T1), and 3 days of temperature recovery (Temperature Recover, Tr).
4. Killing, fixed and save: place the sample in with the concentration of 50% FAA fixed liquid centrifuge tube(Biosharp, http://www.biosharp.cn/index/product/index/language/cn.htm), with pump out excess air centrifugal tube. Later sample observation was commissioned by Shanghai Zhuocai Biotechnology Co., Ltd. (http://zcibio.com/Default.aspx).
5. Observation: After the paraffin section preparation was conducted by the biotechnology company, observations were made using a NIKON ECLIPSE E100 optical microscope equipped with a NIKON DS-U3 imaging system. Subsequently, the Pannoramic MIDI tissue slide digital scanner was used to scan and photograph the samples to obtain image data.

### 2.3 Project measurement

#### 2.3.1 Determination of seedling stage related indexes

When the phenotype-related indexes of maize seedling stage were determined, we selected fixed plants under different treatments, and the second fully unfolded leaf was selected for the determination of leaf related indexes. When measuring the physiological indexes of maize seedlings, we chose to sample 63 maize plants at the same time. Maize leaves are used to measure chlorophyll content and relative electric conductivity. The aboveground parts of plants are used to measure other physiological indicators.

Phenotypic trait measurement method:

1. Plant height (cm): the height from the surface of the soil to the top.
2. Stem diameter (mm): the width of the base of the stem.
3. Leaf length (cm): The length from the base of the blade to the tip of the second leaf (fully unfolded leaf) is measured with a straight ruler.
4. Leaf width (cm): Measure the width of the widest part of the second blade (fully unfolded leaf) with a straight ruler.
5. Leaf area (cm^2^): leaf area = leaf length × leaf width ×0.75

Physiological index measurement method (Wang et al., 2023):

1. Determination of chlorophyll content in leaves (Guo et al., 2024)
  - Take fresh plant leaves, wipe the dirt on the surface of the tissue, cut them into pieces (remove the midvein), and mix them well.
  - Weigh 0.2g of cut fresh sample, add 10ml and 95% ethanol into 3 parts, avoid light until tissue turns white.
  - Pour the chloroplast pigment extract into a colorimetric cup with a light diameter of 1cm. The absorbance was determined with 95% ethanol as blank at the wavelength of 665nm and 649nm.
  - Substitute the measured light absorption value into the following formula: C_a_=13.95A_665_-6.88A_649_; C_b_=24.96A_649_-7.32A_665_. From this, the concentrations of chlorophyll a and chlorophyll b (C_a_, C_b_:mg/L) can be obtained, and the sum of the two is the concentration of total chlorophyll.
  - The chlorophyll content in plant tissues can be further calculated according to the following formula: chlorophyll content (mg/g)=[chlorophyll concentration × extraction liquid volume × dilution ratio]/fresh weight of the sample.
2. Measurement of relative conductivity (Hu, 2024): Rinse the fresh leaves with deionized water and dry the surface moisture. The veins were removed, the leaves were cut and divided into three clean test tubes. Each branch was weighed with 0.2g, and 10ml of distilled water was added. After standing for 20h, R1 was determined by conductivity meter, and R2 was determined by boiling water bath for 30min. The conductivity formula is as follows: Conductivity (%)=(R_1_/R_2_) x 100.
3. Determination of physiological indexes: CAT activity was determined by ammonium molybdate colorimetric method, SOD activity was determined by nitrogen blue tetrazolium photochemical reduction method, POD activity was determined by guaiacol method, MDA content was determined by thiobarbituric acid method, and Pro content was determined by indanhydrin chromogenic method. All the physiological indicators were determined using detection kits. POD activity detection kits were purchased from Beijing Solaibao Technology Co., LTD., and other reagents were purchased from Suzhou Keming Biotechnology Co., LTD. Three biological replicates were performed for all indicators.
4. Photosynthetic measurement: The photosynthetic physiological indexes of the second leaf of maize seedlings were measured by Li-6800 photosynthetic analyzer (Luo et al.).

#### 2.3.2 Leaf cell phenotype determination

There were a total of three slices for each treatment of the seedling leaves, and the CaseViewer software was used to intercept two fields of view at 80.0 pixels, so that a total of six fields were captured in one treatment. The acquired field of view was measured with Image J software for upper cuticle thickness, lower cuticle thickness, upper epidermal thickness, lower epidermal thickness, and leaf thickness, with 5 replicates for each field of view (Sidhu and Schneider, 2024).

### 2.4 Data analysis

(1) Descriptive statistics

Microsoft Excel 2021 software was used to calculate the amplitude, mean± standard deviation and coefficient of variation of phenotypic traits and physiological indexes of 63 maize varieties (Zheng et al., 2023).

(2) Analysis of variance

Multivariate analysis of variance was performed using IBM SPSS Statistics 27.0.1 to analyze the relationship between varieties, treatments, varieties and treatments (Tang et al., 2021).

(3) Correlation analysis

Using Origin 2021 software, Correlation analysis using Correlation Plot plug-in, Plot heatmaps of various indicators under CK and low-temperature treatment at the 0.01 and 0.05 levels (person method) (Stansluos et al., 2023).

(4) Principal component analysis (PCA)

IBM SPSS Statistics 27.0.1 software was used for principal component analysis, and maximum variance method was used for rotation method (Dasheng et al., 2024).

(5) Calculation of membership function values


(1) $$\begin{array}{c} \text{Membership function value}:\text{μ}({\text{X}}_{\text{i}})=\frac{X_{i}-X_{min}}{X_{max}-X_{min}},\text{ i}\\ =1,2,3\ldots \ldots ,\text{n} \end{array}$$

(2) $$\text{Weight}:W_{i}=\frac{CVi}{\sum_{i=1}^{n}CVi}$$

(3) $$\text{Comprehensive evaluation}:\text{D}=\sum_{i=1}^{n}[\mu (X_{i})\times W_{i}]$$

In the above formula, $X_{i}$ is the index measurement value; $X_{min}\;and\;\;X_{max}$ are the minimum and maximum values of a certain index of maize varieties respectively; CVi is the coefficient of variation, and the weight of each indicator is the sum of the coefficient of variation divided by the coefficient of variation.

(6) Cluster analysis

SPSS software was used for systematic cluster analysis, ward method was adopted, and square Euclidean distance was used for genetic distance (Strauss and von Maltitz, 2017).

(7) Comparative analysis of differences, comparative analysis of photosynthetic physiology, and comparative analysis of cellular phenotypes were all performed using Prism software for analysis and graphing.

## 3 Results and analysis

### 3.1 Performance of seedlings under low temperature treatment

Based on observations of 63 varieties under low-temperature stress, this experiment classified the cold tolerance of these varieties. The classification was guided by Li Zhao’s low-temperature stress methodology and adjusted according to the results obtained in this study (Li, 2017). After 3 days of low-temperature stress (**Table 2**), 15 maize varieties showed no adverse effects, with the highest frequency (17 varieties) occurring at cold stress grade III. After 6 days of low-temperature stress, 9 maize varieties remained unaffected, while the majority experienced cold damage at grade II, accounting for 30.16%. During the continuous low temperature stress, most maize varieties were subjected to different degrees of low temperature chilling injury.

**Table 2:** Low temperature classification of 63 maize varieties.

Rank	Low temperature expression	Trait			
		T1	Proportion	T2	Proportion
V	The plant is healthy, the leaves grow normally, green and shiny, basically unaffected.	3, 9, 10, 11, 12, 22, 29, 30, 33, 35, 54, 59, 60, 63	14 (22.22%)	3, 9, 10, 11, 12, 29, 35, 60, 63	9 (14.29%)
IV	The first leaf is slightly wilted, the second and third leaves grow normally, and the heart leaves are normal	1, 2, 8, 14, 16, 17, 18, 31, 32, 50, 51, 52, 55	13 (20.64%)	30, 31, 33, 50, 54, 59, 17, 32, 18	9 (14.29%)
III	The edge of the first leaf is wilted, the edge of the second leaf is wilted, the third leaf is partially wilted, and the heart leaf is slightly wilted.	4, 5, 15, 21, 24, 25, 26, 27, 28, 40, 41, 43, 44, 46, 48, 49, 57	17 (26.98%)	5, 8, 14, 15, 21, 22, 24, 27, 28, 41, 44, 46, 48, 49, 51, 52, 53, 55, 61	17 (26.98%)
II	The first leaf is wilted, the second leaf and the third leaf are partially or mostly wilted, the core leaf is slightly frozen, and the plant can resume growth.	7, 13, 19, 23, 38, 39, 42, 45, 53, 56, 58, 61, 62	13 (20.64%)	1, 2, 4, 7, 13, 16, 19, 23, 25, 26, 38, 42, 43, 45, 53, 56, 57, 58, 62	19 (30.15%)
I	All the leaves and the heart leaf were damaged by freezing, the leaves lost water and browned, wrinkled and sagging, the heart leaf was seriously frozen, and there were cold damage marks.	6, 20, 34, 36, 37, 47	6 (9.52%)	3, 6, 20, 34, 36, 37, 39, 40, 47	9 (14.29%)

### 3.2 Changes of phenotypic characters and physiological indexes of seedlings under low temperature treatment

The phenotypic traits and physiological indicators of 63 maize varieties at the seedling stage were compared and analyzed (**Table 3**). Under control conditions (CK), the coefficient of variation ranged from 12.57% to 46.88%, indicating significant differences among the varieties. Specifically, the stem diameter exhibited the lowest variability (12.57%), while the MDA content showed the highest variability (46.88%). Under T1 and T2 treatments, the coefficients of variation for leaf width and relative conductivity were the lowest, at 11.19% and 8.43%, respectively. Conversely, the MDA content exhibited the highest variability, with coefficients of 47.67% and 45.72% under T1 and T2, respectively. This indicates that while MDA content varied significantly among different maize varieties under low-temperature stress, the changes in leaf width and relative conductivity were relatively consistent across varieties. Under the Tr treatment, the coefficient of variation for stem diameter was the lowest, while that for CAT activity was the highest. This indicates that during the normal temperature recovery phase following low-temperature stress, there was significant variability among maize varieties in their ability to remove hydrogen peroxide, as reflected by CAT activity. In contrast, the stem diameter remained relatively stable. Under each treatment condition, significant variability was observed in Pro, CAT, SOD, and POD levels, indicating substantial differences among the different varieties. These results suggest that low temperatures have a pronounced effect on enzyme activity. Additionally, the results of the variance analysis (**Table 4**) revealed no significant differences in leaf length between treatments or interactions. However, other trait indices exhibited highly significant differences among varieties, treatments, and their interactions. This suggests that low-temperature stress had minimal impact on leaf length during the seedling stage of maize but significantly affected other traits. Furthermore, it indicates that cold resistance varies considerably among different maize varieties.

**Table 3:** Changes of phenotypic traits and physiological indexes of different maize varieties.

Index	Project	Treat time			
		CK	T1	T2	Tr
Plant height(cm)	range	15.00∽45.00	15.00∽45.70	17.40∽46.00	17.00∽51.00
	mean ± stdev	29.21 ± 6.40	30.27 ± 6.58	30.88 ± 6.41	36.88 ± 7.54
	cv(%)	21.93	21.75	20.76	20.45
Stem thick(mm)	range	2.23∽4.74	2.04∽4.67	1.82∽4.84	2.61∽5.32
	mean ± stdev	3.59 ± 0.45	3.78 ± 0.46	3.82 ± 0.48	4.29 ± 0.52
	cv(%)	12.57	12.30	12.59	12.15
Leaf length(cm)	range	8.50∽20.70	8.30∽20.70	8.20∽21.00	6.50∽20.50
	mean ± stdev	15.13 ± 2.27	15.23 ± 2.33	15.27 ± 2.28	15.03 ± 2.33
	cv(%)	15.02	15.31	14.93	15.48
Leaf width(cm)	range	0.90∽2.40	1.00∽2.20	1.00∽2.00	0.40∽2.00
	mean ± stdev	1.62 ± 0.22	1.61 ± 0.18	1.42 ± 0.24	1.32 ± 0.30
	cv(%)	13.70	11.19	17.19	23.03
Leaf area(cm^2^)	range	7.65∽31.50	8.93∽29.37	7.56∽26.79	2.44∽25.65
	mean ± stdev	18.53 ± 4.14	18.42 ± 3.83	16.38 ± 4.16	15.07 ± 4.70
	cv(%)	22.32	20.78	25.42	31.21
Chlorophyll content(mg/g)	range	1.44∽6.13	1.21∽3.45	0.83∽3.84	0.94∽3.52
	mean ± stdev	2.61 ± 0.62	2.16 ± 0.40	2.04 ± 0.60	1.91 ± 0.55
	cv(%)	23.82	18.50	29.32	28.77
Relative electrolytic leakage(%)	range	25.87∽89.62	45.64∽94.94	62.78∽99.80	20.46∽92.65
	mean ± stdev	64.41 ± 13.95	76.95 ± 10.10	87.66 ± 7.39	66.89 ± 15.89
	cv(%)	21.66	13.13	8.43	23.75
MDA(nmol/g FW)	range	1.81∽18.83	2.58∽29.67	2.32∽19.35	3.61∽14.71
	mean ± stdev	7.34 ± 3.44	11.34 ± 5.41	8.02 ± 3.67	8.99 ± 2.35
	cv(%)	46.88	47.67	45.72	26.10
Pro(µg/g FW)	range	29.01∽90.84	46.87∽125.01	30.36∽156.88	19.99∽142.87
	mean ± stdev	56.80 ± 14.15	82.02 ± 14.96	79.86 ± 24.42	79.81 ± 28.77
	cv(%)	24.92	18.24	30.57	36.05
CAT(μmol/min/g FW)	range	1.10∽33.41	5.50∽35.68	6.08∽39.01	2.43∽26.20
	mean ± stdev	18.96 ± 7.48	18.33 ± 6.39	21.51 ± 6.65	14.22 ± 6.67
	cv(%)	39.47	34.84	30.90	46.86
SOD(U/g FW)	range	164.71∽1745.45	160.73∽1798.90	399.57∽2300.84	300.17∽1745.45
	mean ± stdev	955.50 ± 408.32	1281.17 ± 359.35	1217.70 ± 376.86	1027.40 ± 354.90
	cv(%)	42.73	28.05	30.95	34.54
POD(U/g FW)	range	2156.00∽13622.00	4557.00∽17199.00	2940.00∽14063.00	2940.00∽15092.00
	mean ± stdev	5982.15 ± 1919.06	8930.96 ± 2339.85	8680.00 ± 2291.10	9469.19 ± 2284.27
	cv(%)	32.08	26.20	26.40	24.12

**Table 4:** Analysis of variance of maize varieties.

Index	PH	ST	LL	LW	LA	Chl	REL	MDA	Pro	CAT	SOD	POD
Variety	188.30**	39.24**	39.29**	23.30**	39.24**	40.00**	55.28**	101.00**	42.03**	39.74**	60.76**	128.32**
Treat	836.80**	313.58**	1.64	270.47**	135.06**	425.96**	1494.52**	584.93**	555.48**	516.09**	331.34**	2415.52**
Variety×Treat	3.08**	1.86**	0.19	4.89**	2.52**	13.24**	21.72**	25.08**	21.89**	40.11**	19.16**	59.03**

In the table, PH, ST, LL, LW, LA, Chl, REL, MDA, Pro, CAT, SOD and POD indicate plant height, stem diameter, leaf length, leaf width, leaf area, chlorophyll content, relative conductivity, malondialdehyde content, proline content, catalase, superoxide dismutase and peroxidase, respectively. The same as in the following table.

“**” indicates an extremely significant difference.

### 3.3 Comparative analysis of differences in phenotypic traits

**Figure 3:**
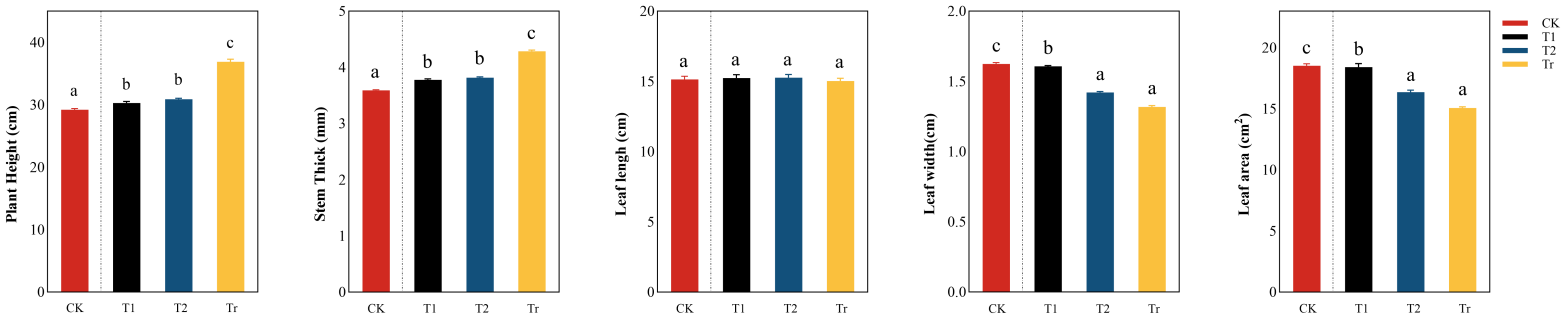
Comparative analysis of differences in phenotypic traits in seedlings “a, b, c, d” are letter codes from multiple comparisons, indicating significance.

Phenotypic traits were analyzed for change trend (**Figure 3**). It can be seen from the figure that plant height and stem diameter generally showed an upward trend under low temperature stress. During low-temperature stress, CK showed extremely significant differences with low-temperature stress, while there was no significant difference between T1 and T2. In the Tr stage, plant height and stem diameter increased significantly, and there was a significant relationship with T1 and T2, indicating that low temperature stress signiLficantly inhibited the growth of plant height and stem diameter. There was no significant difference in leaf length between treatments, but leaf width and leaf area showed a decreasing trend during the low temperature stress period, indicating that the effect of low temperature stress on leaves was significant. In addition, there was no significant difference between CK and T1, indicating that the changes in leaf width and leaf area were small after 3 days of low temperature stress.

### 3.4 Comparative analysis of differences in physiological indicators

**Figure 4:**
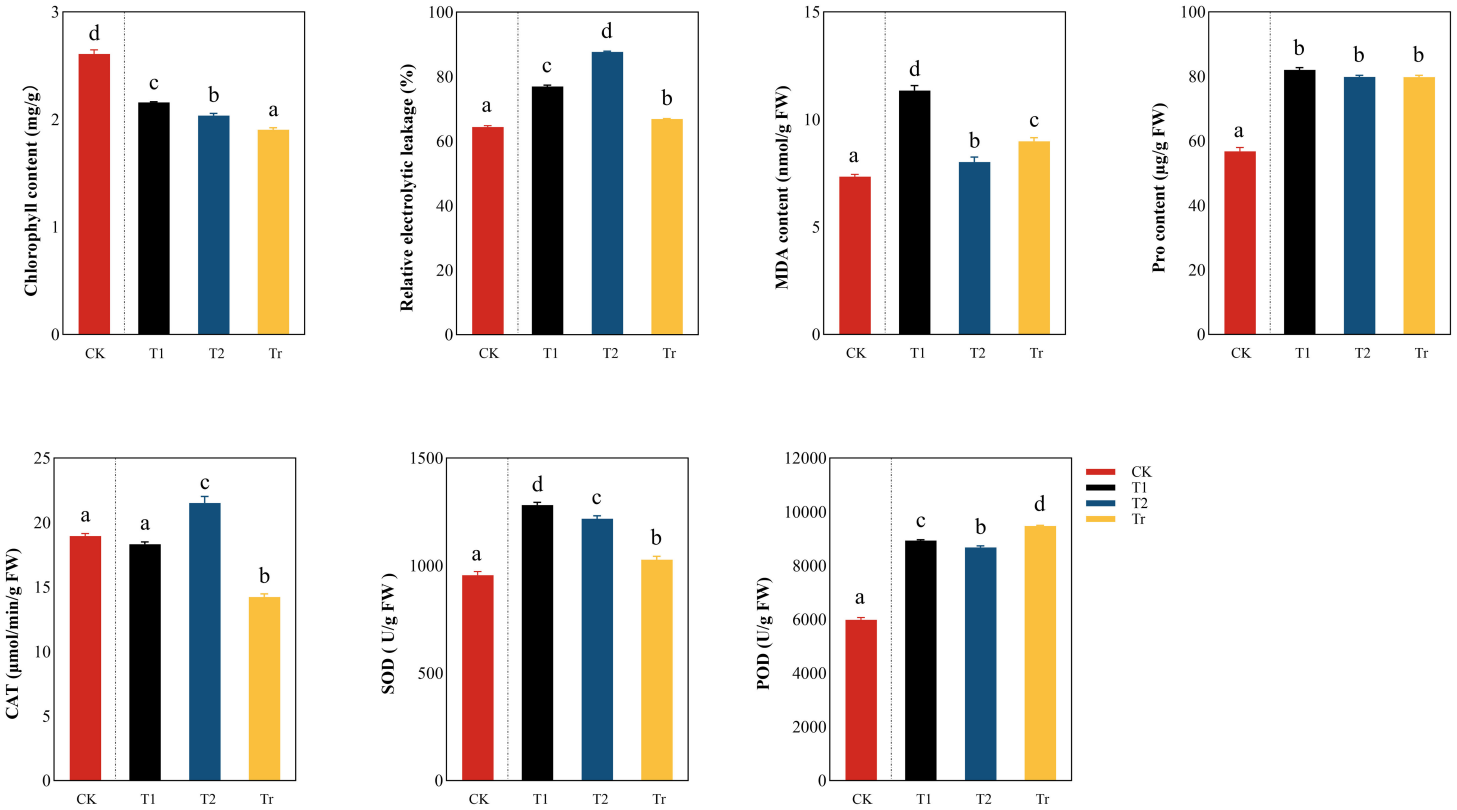
Comparison analysis of differences in physiological indicators of seedlings “a, b, c, d” are letter codes from multiple comparisons, indicating significance.

During low temperature stress (**Figure 4**), the chlorophyll content showed a decreasing trend, In the T1 stage, the chlorophyll content decreased significantly (the decrease range was 17.26%), indicating that the degree of damage to the leaves was greater due to low temperature, resulting in a sharp decrease in chlorophyll content. At the T2 stage, the chlorophyll content did not decrease significantly (the decrease was 5.69%), and it was speculated that the damage of seedlings at this stage was more serious, which affected photosynthesis. The relative conductivity and MDA content showed a significant upward trend in the T1 stage (the relative conductivity increased by 19.47% and the MDA content increased by 54.49%). However, in the T2 stage, the MDA content decreases.

The increase trend of Pro content was obvious in the T1 stage (44.39%), but there was no significant difference in the low temperature stress period and the Tr stage. This results indicate that low temperature will lead to the damage of the cell membrane and the increase of cell membrane permeability, resulting in the extravasation of substances and electrolytes in the cell membrane, and the seedlings will accumulate a large amount of proline in order to resist the damage caused by low temperature stress. In the T2 and Tr stages, the difference in Pro content is not significant, indicating that at the T2 stage, the Pro content has already reached the threshold.

In the T1 stage, SOD and POD showed an upward trend and decreased in the T2 stage, but in the Tr stage, POD showed a significant upward trend, and SOD still showed a significant downward trend. In addition, there was no significant difference between CK and T1 in CAT but its activity increased significantly in the T2 stage and decreased significantly in the Tr stage. This indicates that when seedlings resist low-temperature stress, their antioxidant enzymes will rise significantly.

### 3.5 Correlation analysis of maize seedling stage

**Figure 5:**
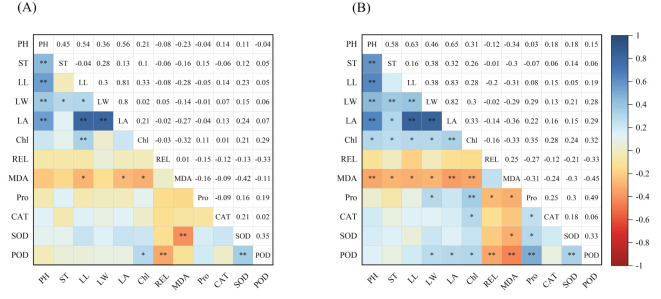
**(A, B)** In the table, PH, ST, LL, LW, LA, Chl, REL, MDA, Pro, CAT, SOD and POD indicate plant height, stem diameter, leaf length, leaf width, leaf area, chlorophyll content, relative conductivity, malondialdehyde content, proline content, catalase, superoxide dismutase and peroxidase, respectively. “*” indicates a significant difference between indicators, and “**” indicates an extremely significant difference.

Correlation analysis of the 12 traits (**Figure 5**) revealed that under control conditions (**Figure 5**), plant height exhibited significant positive correlations with stem diameter, leaf length, leaf width, and leaf area. Additionally, stem diameter was positively correlated with leaf width, and leaf length showed positive correlations with leaf width, leaf area, and chlorophyll content. Chlorophyll content showed significant correlations with MDA content and POD activity. Relative conductivity was only significantly negatively correlated with POD activity, while SOD activity exhibited a highly significant positive correlation with POD activity. Under low-temperature stress conditions (**Figure 5**), plant height exhibited positive correlations with stem diameter, leaf length, leaf width, leaf area, and chlorophyll content. Stem diameter was positively correlated with leaf width, leaf area, and chlorophyll content. Leaf length and leaf width also showed positive correlations with chlorophyll content. Chlorophyll content was negatively correlated with MDA content but positively correlated with proline content as well as CAT and POD activities. Relative conductivity exhibited negative correlations with proline content and POD activity. Proline content showed positive correlations with the antioxidant enzymes, specifically SOD and POD, which were also positively correlated with each other. The correlation patterns of all traits differed under control (CK) and low-temperature stress conditions, indicating that low-temperature stress significantly influenced the changes in each trait.

### 3.6 Principal component analysis of maize seedling stage

Principal component analysis (**Table 5**) was carried out for each index of maize seedling stage. Seven indexes were extracted according to the standard that the cumulative contribution rate was greater than 85%, namely, leaf area, SOD activity, stem diameter, relative conductivity, proline content, CAT activity and chlorophyll. The contribution rate from large to small is 33.13%, 16.67%, 9.36%, 8.23%, 7.39%, 6.79%, 5.57%, and the cumulative contribution rate is 87.12%. The first principal component had an eigenvalue variance of 3.975, showing the strongest correlation with leaf area—a phenotypic index—indicating that low-temperature treatment had the most significant effect on leaf area, with an eigenvalue of 0.969. The second principal component exhibited a variance of 2.000 and was most strongly correlated with SOD activity, an enzyme activity index, suggesting that SOD plays a crucial role in enhancing maize’s cold resistance, with an eigenvalue of 0.827. The third principal component, with a variance of 1.123, was most closely related to stem diameter (eigenvalue = 0.995), indicating the impact of low temperatures on this trait. The fourth principal component had a variance of 0.987 and showed the smallest characteristic value for relative conductivity (-0.94), highlighting its sensitivity to low temperatures. The fifth principal component, with a variance of 0.887, was associated with proline content, a physiological index (characteristic value = 0.798), further indicating the influence of low-temperature treatment on proline levels. The sixth principal component, corresponding to CAT activity, had a variance of 0.912, suggesting that increased CAT activity contributes to enhanced cold resistance. Lastly, the seventh principal component, with a variance of 0.668, was linked to chlorophyll content, a photosynthesis-related index (characteristic value = 0.887), indicating that low temperatures affect maize leaf photosynthesis. In summary, leaf area, SOD activity, stem diameter, relative electrical conductivity, proline content, CAT activity, and chlorophyll content can serve as key evaluation indices for assessing the cold resistance of maize at the seedling stage.

**Table 5:** Principal component analysis of each index.

The measured index feature vector load	Principal component						
	PC1	PC2	PC3	PC4	PC5	PC6	PC7
PH	0.588	0.094	0.565	0.058	-0.234	0.235	0.191
ST	0.104	0.105	0.955	0.023	0.054	-0.042	0.053
LL	0.827	0.135	-0.087	0.13	-0.25	0.162	0.308
LW	0.787	0.079	0.308	-0.118	0.398	-0.057	-0.147
LA	0.969	0.141	0.102	0.022	0.052	0.073	0.105
Chl	0.195	0.217	0.108	0.056	0.199	-0.023	0.887
REL	0.007	-0.062	-0.055	-0.94	-0.026	-0.157	-0.022
MDA	-0.146	-0.785	-0.156	0.032	0.016	-0.244	-0.247
Pro	-0.034	0.226	-0.006	0.116	0.798	0.24	0.211
CAT	0.144	0.094	0.003	0.119	0.166	0.912	-0.018
SOD	0.111	0.827	0.049	0.165	0.224	-0.034	0.029
POD	0.141	0.495	-0.071	0.548	0.392	-0.129	0.129
Eigen value	3.975	2.000	1.123	0.987	0.887	0.815	0.668
Contribution/%	33.127	16.666	9.356	8.228	7.388	6.788	5.566
Cumulative contribution rate/%	33.127	49.793	59.149	67.377	74.765	81.553	87.119

### 3.7 D value and cluster analysis of each index of maize seedling stage

**Figure 6:**
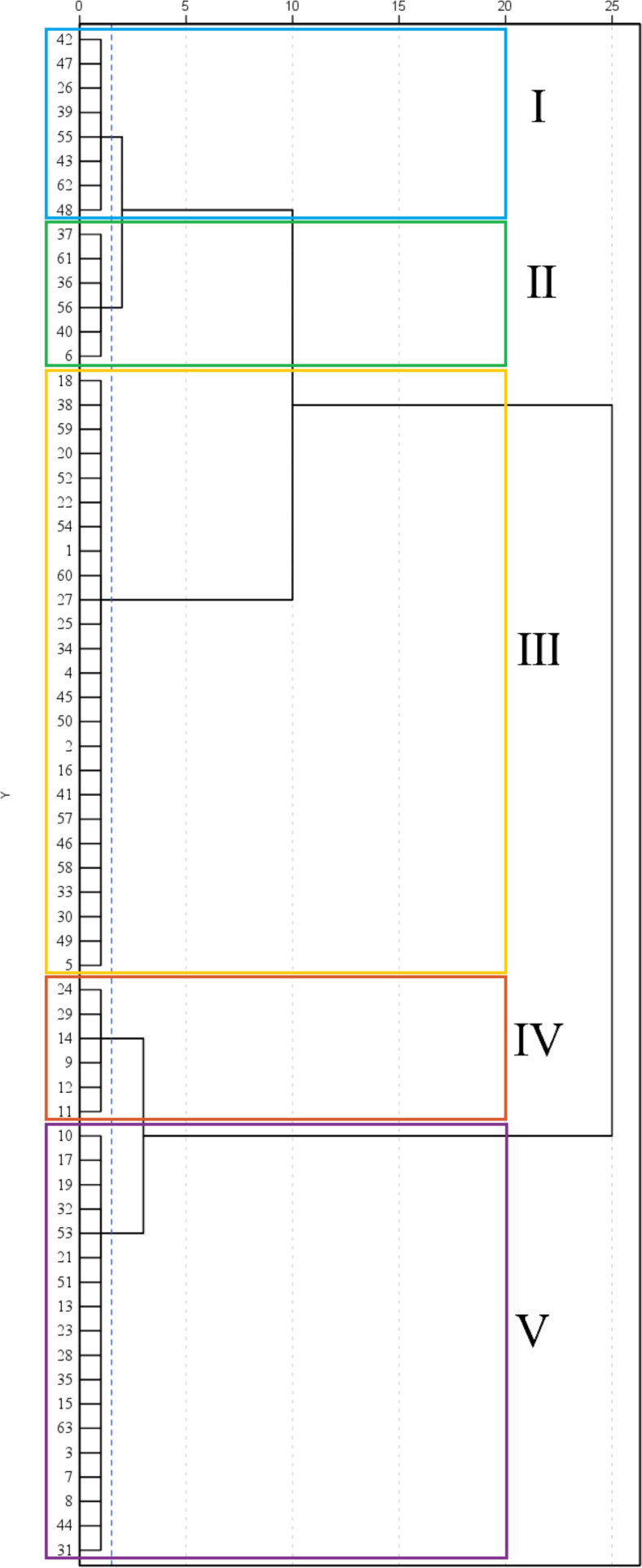
Cluster analysis of 63 maize varieties. Different colors in the figure distinguish between the five groups, with Group I represented by blue, Group II by green, Group III by yellow, Group IV by red, and Group V by purple. The dotted axes in the figure represent radial values, indicating Euclidean distances.

The comprehensive evaluation D-value was calculated using Equations 1-3, and the 63 maize materials were ranked accordingly (**Table 6**). Cluster analysis using the D-value (**Table 7**; **Figure 6**) categorized the 63 varieties into five groups based on a Euclidean distance threshold of 1.5: extremely strong cold resistance, strong cold resistance, moderate cold resistance, weak cold resistance, and sensitive to cold.

**Table 6:** D-value ranking and group division of 63 maize varieties.

Code	D Value	Group	Code	D Value	Group	Code	D Value	Group
11	0.686	IV	21	0.572	V	30	0.491	III
12	0.672	IV	53	0.568	V	49	0.490	III
9	0.667	IV	32	0.568	V	33	0.487	III
24	0.655	IV	18	0.548	III	46	0.486	III
29	0.652	IV	38	0.547	III	58	0.485	III
14	0.637	IV	59	0.545	III	57	0.475	III
19	0.623	V	20	0.537	III	41	0.474	III
10	0.612	V	52	0.535	III	62	0.453	I
17	0.610	V	22	0.532	III	43	0.451	I
35	0.602	V	54	0.532	III	48	0.440	I
28	0.596	V	1	0.530	III	55	0.430	I
23	0.594	V	60	0.529	III	39	0.424	I
15	0.588	V	27	0.522	III	47	0.415	I
63	0.588	V	50	0.512	III	42	0.415	I
3	0.587	V	45	0.511	III	26	0.410	I
7	0.586	V	2	0.509	III	36	0.391	II
31	0.582	V	16	0.507	III	56	0.390	II
8	0.579	V	34	0.505	III	40	0.385	II
44	0.577	V	25	0.504	III	61	0.363	II
13	0.574	V	4	0.503	III	37	0.361	II
51	0.572	V	5	0.495	III	6	0.323	II

**Table 7:** Comparison of the mean values of each index of different groups and the division of D values.

Project	Group				
	I	II	III	IV	V
PH	28.63	22.18	30.09	38.00	36.75
ST	3.91	3.29	3.86	4.15	3.96
LL	13.88	12.61	14.85	16.60	16.55
LW	1.35	1.28	1.48	1.71	1.58
LA	14.06	12.14	16.46	21.19	19.58
Chl	1.97	1.73	2.14	2.63	2.31
REL	75.67	74.73	74.45	74.68	72.09
MDA	9.22	10.59	8.92	8.42	8.41
Pro	63.60	67.91	74.89	83.68	78.37
CAT	16.25	15.94	17.98	20.16	19.66
SOD	996.30	833.82	1153.92	1317.30	1159.05
POD	7311.72	7448.68	8224.49	9210.64	8703.85
D value	0.41-0.45	0.32-0.39	0.47-0.55	0.64-0.69	0.57-0.62
Proportion	8 (12.70%)	6 (9.52%)	25 (39.68)	6 (9.52%)	18 (28.57%)

Group IV contained 6 varieties (9.52%) and exhibited the highest values for all traits except relative conductivity and MDA content, with a D-value of 0.66. This group showed the largest phenotypic traits, highest antioxidant enzyme activity, and lowest MDA content, The results indicated that the maize varieties in this group had a strong ability to resist low temperatures Thus, this group was classified as having extremely strong cold tolerance. Group V included 18 varieties (28.57%), with the second-highest average trait values apart from the lowest relative conductivity and MDA content, and a D-value of 0.59. The phenotypic traits and antioxidant enzyme activity of this group ranked second, leading to its classification as having strong cold tolerance. A total of 25 groups were found in group III, accounting for 39.68%, and the proportion was the largest among the five categories, with a D value of 0.51, and the relative conductivity of this group was the lowest, indicating that the cell membrane damage of seedlings was small, and other traits ranked third, and this group was divided into medium type of cold tolerance. Group I consisted of 8 varieties (12.70%) with a D-value of 0.43. This group exhibited the highest relative conductivity, second-highest MDA content, lowest proline content and POD activity, and fourth-ranked average values for other traits. The results indicated that the cell membrane of the seedlings in this group was damaged and the electrolyte extravasation was serious, so they were classified as cold-tolerant and weak. The phenotypic traits and antioxidant enzymes of group II. were the lowest among the five categories, indicating that low temperature significantly affected the growth of maize seedlings in this group, and the ability to resist low temperature was poor, and the seedlings were subjected to certain frost damage, so this group was classified as cold-tolerant and sensitive. According to the above analysis of the differences in the indexes of each taxa, the cold tolerance of the taxa was in the order of IV.>V.> III.> I.>II. According to the results of comprehensive cluster analysis and D-value ranking, the top five varieties with D-value were successively 11(Jiuyang 818)>12(Youqi 666)>9(Simon XM1618)>24(Kenyu 90)>29(Aomei 95), and the last five varieties were: 6 (JR288) <37(Gan Xin 2818) <61 (Liangyu 918) <40 (Liangyu 99) <56 (Liangyu 88).

### 3.8 Observation of seedling phenotype and leaf performance of extreme materials

**Figure 7:**
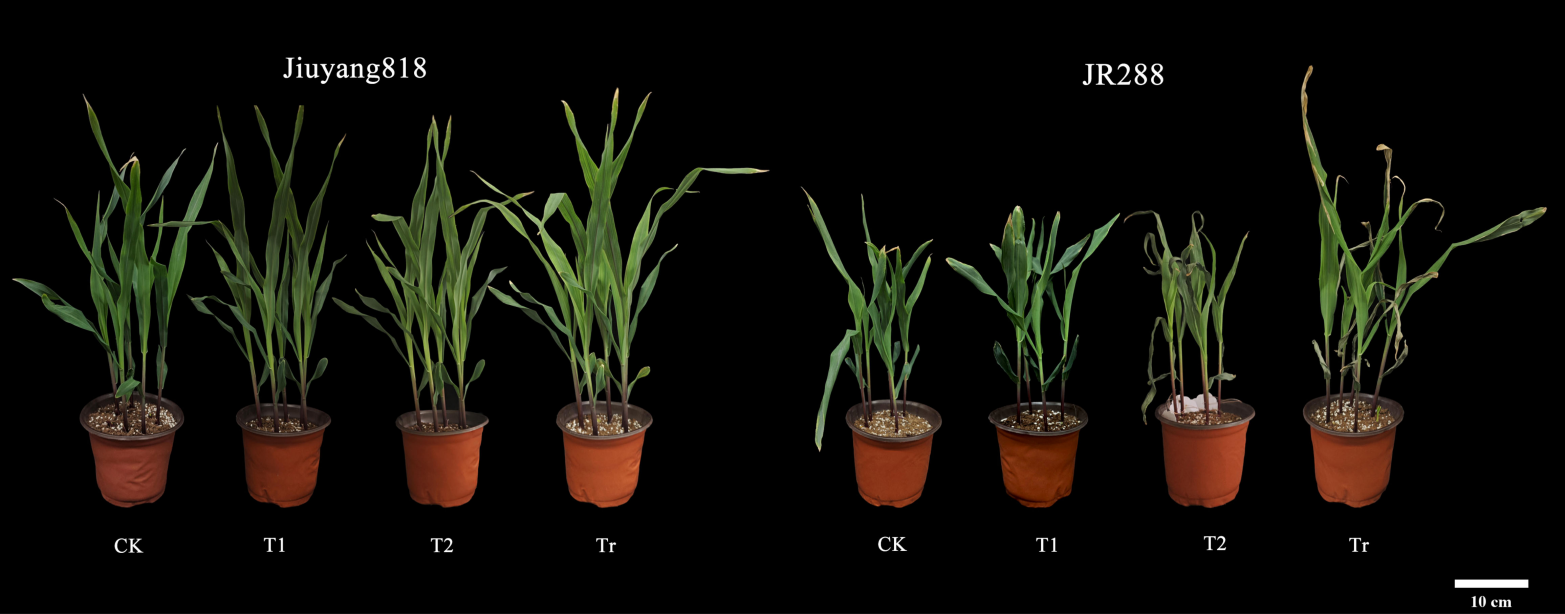
Overall plant performance and leaf observations of extreme maize varieties.

Based on the D-value and cluster analysis results, we observed the leaf phenotypes of two extreme varieties: the highly cold-tolerant variety 11 (Jiuyang 818) and the cold-sensitive variety 6 (JR288). According to the overall observation of maize plants at the seedling stage (**Figure 7**), under control (CK) conditions, Jiuyang 818 exhibited significantly greater plant height and more robust growth compared to JR288. After 3 days of low-temperature stress (T1), no significant differences were observed between the two varieties. However, after 6 days of low-temperature stress, JR288 showed severe wilting, particularly with pronounced cold damage to the first leaf and leaf center. The second leaf displayed noticeable atrophy and clear freeze marks. During the recovery period following low-temperature stress, while JR288 plants showed some degree of recovery, the damage remained significant, with leaves turning yellow and wilting. The results indicated that low temperature stress would lead to water loss and even death of leaves. It affects the normal growth and development and photosynthesis of maize seedlings, and then leads to the inability to grow normally at room temperature recovery stage. Through the observation of two extreme materials, it is further illustrated that there are obvious differences in the ability of different genotypes of maize to withstand low temperature.

### 3.9 Photosynthetic physiological analysis of extreme materials under low temperature stress

**Figure 8:**
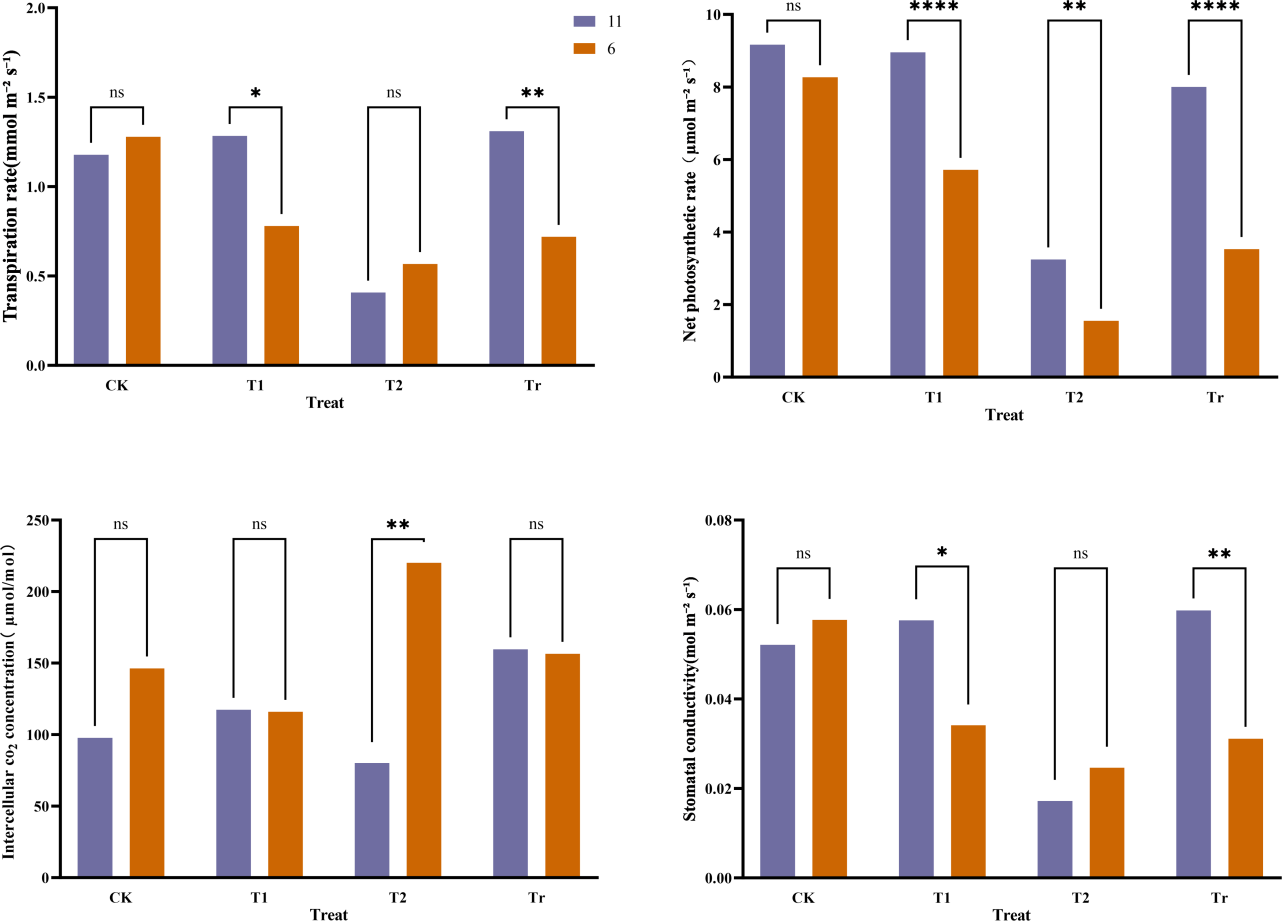
Comparison of photosynthetic physiology of extreme materials. In the legend, "11" and "6" represent the varieties Jiuyang 818 and JR288, respectively. CK, T1, T2, and Tr denote control conditions, three days of low-temperature stress, six days of low-temperature stress, and three days of recovery at normal temperature, respectively “*” indicates a significant difference, “**” and “****” indicate extremely significant differences, and “ns” indicates no significant difference.

The transpiration rate, net photosynthetic rate, intercellular CO_2_ concentration, and stomatal conductance of the second leaf were compared between the two extreme maize varieties. The results (**Figure 8**) indicated that under control (CK) conditions, there were no significant differences in photosynthetic physiology between the two varieties. After 3 days of low-temperature stress (T1), variety No. 11 exhibited significantly higher transpiration rates and stomatal conductance compared to variety No. 6, with a notably higher net photosynthetic rate as well. Following 6 days of low-temperature stress (T2), significant differences emerged in both net photosynthetic rate and intercellular CO_2_ concentration between the two varieties, with variety No. 6 showing a significantly higher intercellular CO_2_ concentration than No. 11. Overall, during the low-temperature stress period, the transpiration rate, intercellular CO_2_ concentration, and stomatal conductance of variety No. 11 initially increased before decreasing, whereas those parameters for variety No. 6 consistently declined. During the recovery phase at room temperature, the transpiration rate, intercellular CO_2_ concentration, and stomatal conductance of variety No. 11 surpassed their CK levels, while variety No. 6 did not fully recover to CK levels except for intercellular CO_2_. Other photosynthetic physiological parameters of variety No. 6 remained significantly lower than those of variety No. 11. These findings indicate that low-temperature stress caused a decrease in the transpiration rate, net photosynthetic rate, and stomatal conductance of the plants, further suggesting that variety No. 11 possesses stronger cold tolerance than variety No. 6.

### 3.10 Changes of leaf cell structure in response to low temperature stress in maize seedlings

**Figure 9:**
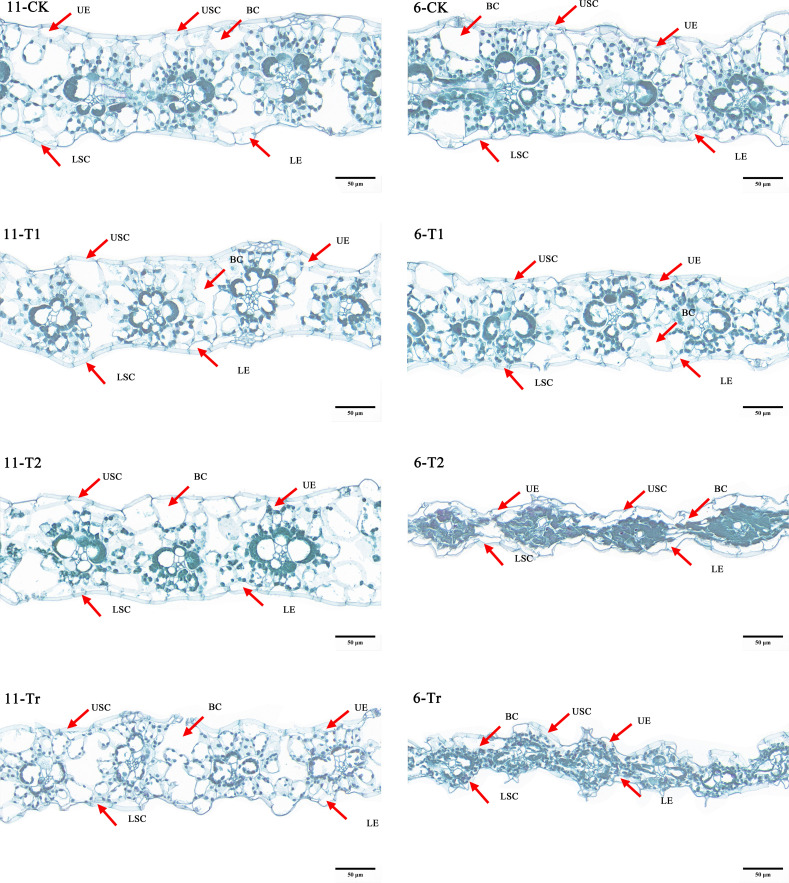
Cell observations of extreme materials. The number 11 in the figure is the very strong cold resistance variety (Jiuyang 818), and the number 6 is the cold sensitive variety (JR288). The observation ratio of all images in the figure is Bar=50µm and 80.0pixel/μm.

The results of paraffin section observations of maize seedlings(**Figure 9**). Under normal temperature conditions (CK), the vascular sheath cells in the leaves of variety 11 (Jiuyang 818) contained a higher density of chloroplasts and appeared darker, indicating superior photosynthetic efficiency. After three days of low-temperature stress (T1), both varieties maintained structurally intact epidermal cells, vesicular cells, and vascular sheath cells, with orderly morphology, but the chloroplasts of variety 6 already show signs of messiness. Following six days of low-temperature stress (T2), the chloroplasts in variety 11 showed minor signs of disorganization. In contrast, variety 6 exhibited evident epidermal cell atrophy, ruptured vesicular cells, and damaged vascular sheath cells. The cellular structure was severely compromised, with cell fluids exuding and chloroplasts becoming unrecognizable. During the recovery phase after low-temperature stress (Tr), the epidermal, mesophyll, and vascular sheath cells of variety 11 showed signs of recovery, with clearly distinguishable morphology. Although the chloroplasts remained intact in shape, their numbers were significantly reduced compared to control conditions, likely due to prolonged low-temperature exposure. In contrast, the recovery of variety 6 was suboptimal; its cellular structure remained disorganized, and the structural integrity of epidermal, vesicular, and vascular sheath cells could not be restored. Observation of cells further explains that cold-resistant materials have a smaller impact on photosynthesis under low-temperature stress and a lesser effect on cell structure.

### 3.11 Effects of low temperature stress on leaf epidermis of maize seedlings

**Figure 10:**
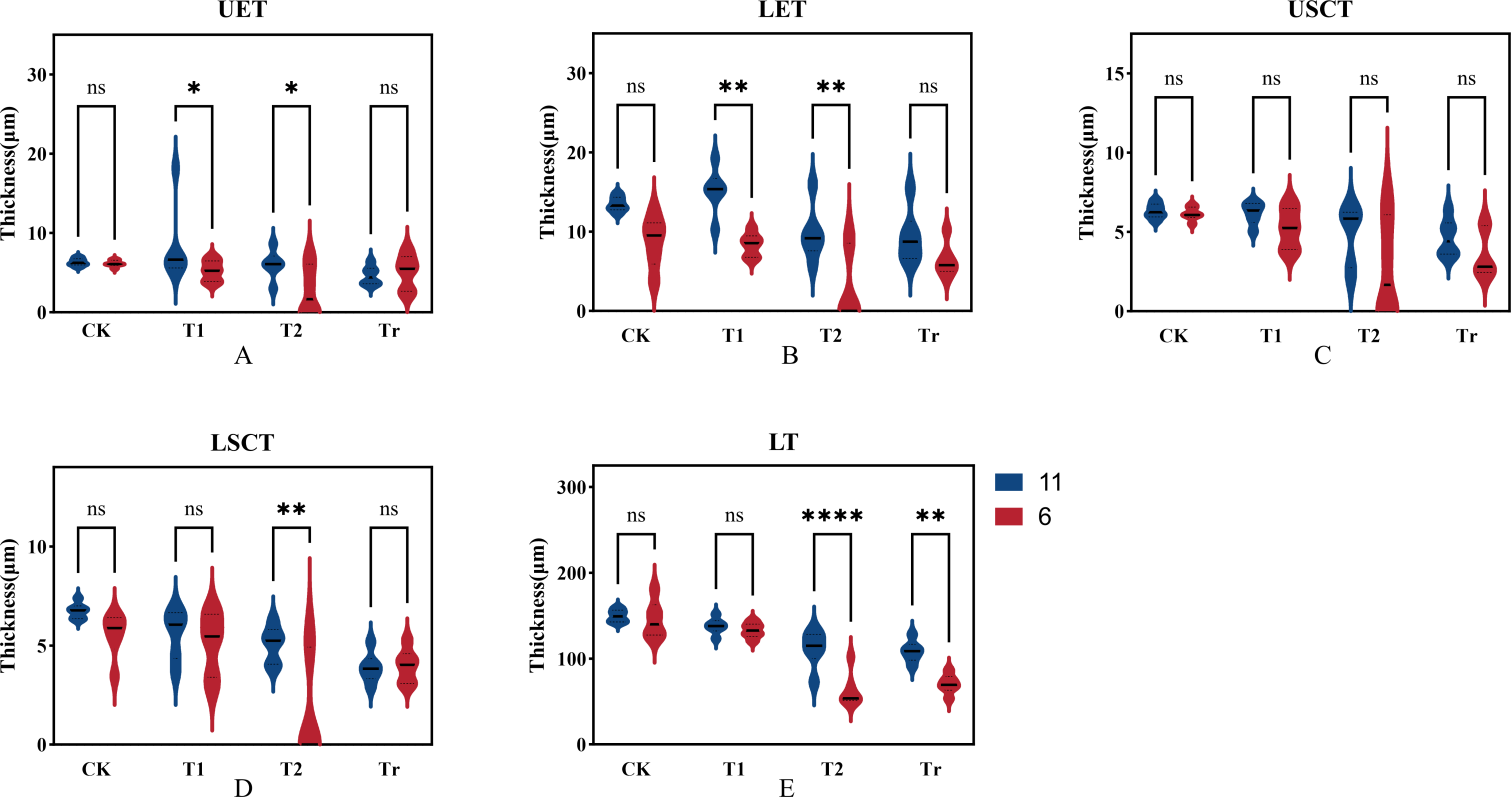
Comparison of cell structure indices in extreme materials. In the figure, UET, LET, USCT, LSCT, and LT represent Upper Epidermal Thickness, Lower Epidermal Thickness, Upper Stratum Corneum Thickness, Lower Stratum Corneum Thickness, and Leaf Thickness, respectively. In the legend, "11" and "6" denote the varieties Jiuyang 818 and JR288, respectively. CK, T1, T2, and Tr indicate control conditions, three days of low-temperature stress, six days of low-temperature stress, and three days of recovery at normal temperature, respectively.

Based on the cellular data obtained from paraffin sections, we compared and analyzed the thickness of the upper and lower stratum corneum, upper and lower epidermis, and overall leaf thickness for both varieties (**Figure 10**). Under normal temperature conditions (CK), variety No. 11 (Jiuyang 818) exhibited greater thickness in the epidermis, stratum corneum, and overall leaf structure compared to variety No. 6 (JR288). After three days of low-temperature stress (T1), the epidermal thickness of variety No. 11 was significantly greater than that of variety No. 6, showing a marked relationship. Compared to control conditions, the epidermal layer thickened while the cuticle layer and overall leaf thickness decreased, It is inferred that plants will resist low temperatures by increasing leaf thickness. For variety No. 6, except for the increase in epidermal thickness, other phenotypic traits showed a downward trend, this indicates that cold-sensitive materials are less able to withstand low temperatures and are more severely damaged. During the recovery phase after low-temperature stress (Tr), there were significant differences in leaf thickness between the two varieties, suggesting that low-temperature stress had a greater effect on leaf thickness. Through the analysis of the phenotypic indexes of the cells, it can be seen that the changes in cell structure are the adaptation of maize seedlings when they resist low temperature stress. At the same time, it was further indicated that the results of cell observation were consistent with those of photosynthetic physiological assays.

## 4 Discussion

### 4.1 Screening for low-temperature tolerant maize varieties is a critical prerequisite for the breeding and identification of cold-tolerant cultivars

Cold tolerance is a physiological response that enables plants to adapt to low-temperature environments. The level of cold tolerance can vary significantly among different varieties of the same crop. Low temperatures can impede plant growth and development, leading to disorders in physiological and biochemical systems. This disruption can result in reduced yields and, consequently, economic losses (Wang et al., 2017). The strength of maize cold tolerance was comprehensively evaluated based on the variation in multiple indices (Cao et al., 2019). Plant cells acclimate to low-temperature stress through modulating the plasma membrane, intracellular osmoprotectants, redox system, and photosynthetic efficiency (Shantappa et al., 2022). In this study, a total of 63 maize varieties were utilized as experimental materials to characterize the seedling stage of maize. The results demonstrated that low-temperature stress significantly inhibited the growth of maize plants, leading to wilting of the leaves and cold damage to the heart leaves (Song et al., 2020). Significant differences were observed in phenotypic traits and physiological indices across varieties, treatments, and their interactions. Notably, there was considerable variation in cold resistance among the maize varieties. In this experiment, low temperature inhibited the growth of seedling height and stem diameter, which had little effect on leaf length, but had the greatest effect on leaf width and leaf area, and showed a decreasing trend (Li et al., 2018; Geng et al., 2004, Gámez-Vázquez et al., 2015). The chlorophyll content determines the photosynthetic performance of plants, and low temperature hinders the photosynthesis of plants, and at the same time leads to physiological changes in maize leaves. In addition, the chlorophyll content showed a decreasing trend after low temperature treatment, which was consistent with the results of Wani et al. (2018) and Ramazan et al. (2021), and there was a significant relationship between chlorophyll content and phenotypic traits, antioxidant enzymes and Pro content. At the same time, chlorophyll content was significantly correlated with phenotypic traits, antioxidant enzymes and Pro content. Low temperature stress affected the phenotype of plants and changed their internal physiology accordingly, so as to resist low temperature chilling injury. The results showed that the activities of antioxidant enzymes were increased in the early stage of low temperature stress, thereby reducing the damage of membrane lipid peroxidation. Meanwhile, the degree of decline in MDA content varies among different maize varieties, maize has developed certain resistance to low temperatures, and there are differences in frost resistance among different maize varieties. This study is consistent with the findings of Salika Ramazan et al., who reported that, under low-temperature treatment, Gurez (a cold-tolerant genotype) exhibited reduced accumulation of stress markers such as hydrogen peroxide (H2O2) and malondialdehyde (MDA), along with a well-developed antioxidant defense system (Ramazan et al., 2021).

Correlation analysis revealed that, under both normal temperature conditions and low-temperature stress, chlorophyll content was positively correlated with leaf length and width. Additionally, MDA levels were negatively correlated with POD activity, while SOD and POD activities exhibited a positive correlation with each other. Low temperature significantly influenced the changes in each characteristic. Principal Component Analysis (PCA) can transform multiple indices into a few comprehensive indicators. Specifically, leaf area, SOD activity, stem diameter, relative conductivity, proline content, CAT activity, and chlorophyll content can serve as key evaluation metrics for assessing the cold resistance of maize during the seedling stage. Clarifying the effects of low-temperature stress on phenotypic traits and physiological indices during the seedling stage can facilitate efficient evaluation of cold tolerance in maize. This understanding can further enrich our knowledge of stress resistance in maize varieties, providing valuable references for establishing low-temperature tolerance indicators and screening for cold-tolerant varieties. Morphological development and biomass accumulation during the seedling stage are crucial factors that influence plant yield and quality. Low temperatures during this early phase can significantly impair the accumulation of organic matter in subsequent growth stages. In this study, a comprehensive evaluation D-value was derived using membership function values, and rankings were established accordingly. Through cluster analysis, the 63 maize varieties were categorized into five groups based on their cold tolerance: extremely strong, strong, moderate, weak, and sensitive. According to the D-value rankings, variety No. 11 exhibited strong cold resistance, whereas variety No. 6 showed the poorest cold tolerance among the tested varieties. Screening maize varieties for low-temperature tolerance during the seedling stage can effectively mitigate the impact of sudden adverse weather conditions and lay a solid foundation for the healthy and normal growth and development of maize in later stages. Identifying varieties with strong low-temperature tolerance is an essential prerequisite for breeding cold-tolerant maize cultivars.

### 4.2 Effects of low temperature stress on maize phenotype and photosynthesis

Maize low-temperature tolerance is a complex quantitative trait influenced by multiple micro-effect genes (Feng et al., 2023). Following exposure to low-temperature stress, distinct differences in both phenotypic and physiological characteristics were observed across different germplasms (Dong et al., 2024). Under low-temperature stress, plant growth was generally inhibited, as evidenced by reductions in plant height (Yang et al., 2018), stem diameter, and leaf area (Bhattacharya, 2022b). To investigate the effects of low temperatures on plant and leaf performance, two extreme maize varieties were selected for observation: the highly cold-tolerant Jiuyang 818 and the cold-sensitive JR288. This study examined the overall performance of plants and leaves under cold exposure. It was found that plants of the extremely cold-tolerant variety Jiuyang 818 exhibited greater vigor and suffered relatively less leaf damage compared to the cold-sensitive variety JR288. Photosynthesis is the primary physiological process affected by low-temperature stress. Cold stress can restrict energy flow and inhibit photosynthetic activity (Fracheboud et al., 2002). The net photosynthetic rate is a direct reflection of the functionality of the photosynthetic system and serves as an indicator of whether the plant’s photosynthetic apparatus is operating normally (Calzadilla et al., 2022). Stomata are the channels through which water vapor and co2 exchange between plants and the atmosphere, influencing both photosynthesis and transpiration (Li et al., 2021). In this experiment, we measured the photosynthetic physiology of two extreme maize varieties. The results indicated that as low-temperature stress prolonged, both varieties experienced continuous declines in transpiration rate, net photosynthetic rate, and stomatal conductance, while intercellular co2 concentration initially decreased and then increased. Notably, the decline in these parameters was significantly less pronounced in Jiuyang 818 compared to JR288. Moreover, the photosynthetic physiological indices of Jiuyang 818 largely returned to control (CK) levels, suggesting that varieties with stronger cold tolerance exhibit lesser impacts on their photosynthetic physiology under low temperatures.

### 4.3 Cytological response of maize leaves to low temperature stress

Cold tolerance refers to a plant’s ability to resist cold damage through long-term adaptation to low-temperature environments. Low temperatures can alter the osmotic pressure within plants, leading to cellular and tissue dehydration (Kazemi-Shahandashti and Maali-Amiri, 2018). Research has demonstrated that in cold-tolerant maize, the leaf epidermis and mesophyll cell layers are thinner, while the cell walls are thicker compared to cold-sensitive maize. Under low-temperature conditions, the leaf thickness and mesophyll cell layer thickness increase in cold-tolerant varieties, whereas these parameters decrease in cold-sensitive strains (Bilska-Kos et al., 2016, 2018). In this study, the epidermal thickness, cuticle thickness, and overall leaf thickness of cold-tolerant maize varieties were found to be greater than those of cold-sensitive varieties. Following low-temperature treatment, the tissue arrangement in the leaves of cold-sensitive maize became notably more disorganized compared to that of cold-tolerant maize, with severe damage observed in the cuticle and epidermis. In contrast, the degree of cell deformation in the leaves of cold-tolerant maize was relatively minor, indicating a lesser impact of low temperatures on cellular structure. These findings further suggest that cellular characteristics and deformation reflect the response mechanisms of maize to low-temperature stress (Leconte et al., 2024). The morphological features of plants, along with their physiological and biochemical characteristics and cellular changes, provide a comprehensive reflection of the plant’s stress resistance mechanisms. These integrated responses offer insights into how plants adapt to and withstand adverse conditions (Wang et al., 2024). Aguilera showed a significant genotypic variation in the susceptibility to and rate of recovery from chilling-dependent photoinhibition of photosynthesis in Zea mays seedlings (Aguilera et al., 1999). This study yielded findings consistent with those of Ricardo Aroca (Aroca et al., 2001), The researcher exposed two maize cultivars with differing cold sensitivities to a 5°C stress treatment for five days, followed by a recovery period at 25°C for three days. The study found. When plants were returned to 25°C, Cold resistant material Z7 leaves quickly recovered photosynthetic activity thus diminishing the potential power for reactive oxygen species generation.

Due to variations in physiological conditions and states, morphological structure indices can be highly variable, even within the same species, often failing to accurately reflect a plant’s stress resistance. Therefore, when evaluating the cold tolerance of maize varieties, it is essential to make a more scientific and comprehensive judgment by considering multiple aspects. In this study, the microscopic structural indices used for assessing cold resistance were based solely on the analysis of seedling leaf microstructures. Determining more precise identification indices for cold resistance will require further research incorporating various comprehensive factors. In conclusion, studying the cellular-level response of maize varieties to low-temperature stress can uncover the mechanisms underlying this stress response. This has important theoretical and practical implications for enhancing maize’s low-temperature stress tolerance, optimizing the utilization of germplasm resources, and breeding new high-quality, cold-tolerant maize varieties.

## 5 Conclusion

Significant differences were observed among the 63 maize varieties, across different treatments, and in their interaction effects, indicating a diverse genetic background within the study materials. Key evaluation indices for assessing cold resistance at the seedling stage include leaf area, SOD activity, stem diameter, relative electrical conductivity, proline content, CAT activity, and chlorophyll content. By integrating membership function analysis with cluster analysis, cold resistance was categorized into five levels: extremely strong, strong, moderate, weak, and sensitive. The performance and photosynthetic physiology of two extreme maize varieties demonstrated that those with strong cold tolerance exhibited superior characteristics compared to cold-sensitive varieties. Furthermore, phenotypic comparisons and cellular-level observations revealed that low-temperature stress had a pronounced impact on maize leaves, highlighting the importance of these metrics in evaluating cold resistance.

## Data Availability

The original contributions presented in the study are included in the article/Supplementary Material. Further inquiries can be directed to the corresponding author.
